# Supplementary material for: Meta-Analysis and Gene Set Enrichment Relative to ER Status Reveal Elevated Activity of MYC and E2F in the “Basal” Breast Cancer Subgroup
Source: PLoS One. 2009 Mar 9;4(3):e4710. doi: 10.1371/journal.pone.0004710 (PMC2650420; doi:10.1371/journal.pone.0004710)
Supplement: Dataset S1 — GSEA results from first screen. The file contains a zipped archive of the HTML results of the second GSEA screen. Reports for enriched or depleted datasets may be accessed from the “index.html” link within the my_analysis.GseaPreranked.1219975950625.rpt.zip ZIP archive. Enrichment plots are available for each of the 50 most enriched and depleted gene sets. (13.32 MB ZIP) [file pone.0004710.s001.zip › my_analysis.GseaPreranked.1219975950625.rpt.zip!//GH_AUTOCRINE_DN.html]

Details for gene set GH\_AUTOCRINE\_DN[GSEA]

|  || Dataset | zSco.ERneg.to.ERpos\_collapsed |
| Phenotype | NoPhenotypeAvailable |
| Upregulated in class | na\_neg |
| GeneSet | GH\_AUTOCRINE\_DN |
| Enrichment Score (ES) | -0.4130844 |
| Normalized Enrichment Score (NES) | -1.9362692 |
| Nominal p-value | 0.0 |
| FDR q-value | 0.013824742 |
| FWER p-Value | 0.34 |
Table: GSEA Results Summary

  

Fig 1: Enrichment plot: GH\_AUTOCRINE\_DN      
 Profile of the Running ES Score & Positions of GeneSet Members on the Rank Ordered List

  

| PROBE | GENE SYMBOL | GENE\_TITLE | RANK IN GENE LIST | RANK METRIC SCORE | RUNNING ES | CORE ENRICHMENT || 1 | PUS7 |  |  | 86 | 5.970 | 0.0177 | No |
| 2 | RHOQ |  |  | 386 | 4.712 | 0.0143 | No |
| 3 | ROD1 |  |  | 855 | 3.700 | -0.0061 | No |
| 4 | DDX39 |  |  | 989 | 3.498 | -0.0020 | No |
| 5 | NETO2 |  |  | 1090 | 3.371 | 0.0041 | No |
| 6 | PODXL |  |  | 1107 | 3.351 | 0.0165 | No |
| 7 | RBM7 |  |  | 1316 | 3.121 | 0.0135 | No |
| 8 | CIT |  |  | 1464 | 2.983 | 0.0145 | No |
| 9 | CSAG2 |  |  | 1579 | 2.870 | 0.0175 | No |
| 10 | HPSE |  |  | 1612 | 2.837 | 0.0266 | No |
| 11 | RELN |  |  | 1673 | 2.780 | 0.0334 | No |
| 12 | PTPRK |  |  | 1740 | 2.731 | 0.0395 | No |
| 13 | TSPYL5 |  |  | 1866 | 2.634 | 0.0407 | No |
| 14 | B4GALT1 |  |  | 2238 | 2.361 | 0.0222 | No |
| 15 | RPL37 |  |  | 2256 | 2.345 | 0.0305 | No |
| 16 | MCF2L |  |  | 2396 | 2.263 | 0.0291 | No |
| 17 | DBN1 |  |  | 2438 | 2.229 | 0.0351 | No |
| 18 | HSPH1 |  |  | 2500 | 2.199 | 0.0394 | No |
| 19 | LXN |  |  | 2669 | 2.106 | 0.0352 | No |
| 20 | ST3GAL2 |  |  | 2897 | 1.987 | 0.0261 | No |
| 21 | TNNT3 |  |  | 2928 | 1.971 | 0.0319 | No |
| 22 | IL1F5 |  |  | 3188 | 1.847 | 0.0198 | No |
| 23 | M-RIP |  |  | 3268 | 1.808 | 0.0211 | No |
| 24 | CD44 |  |  | 3638 | 1.645 | -0.0001 | No |
| 25 | BAMBI |  |  | 3717 | 1.615 | 0.0005 | No |
| 26 | NFYC |  |  | 4165 | 1.445 | -0.0274 | No |
| 27 | TNFRSF4 |  |  | 4211 | 1.427 | -0.0250 | No |
| 28 | CDC42BPA |  |  | 4330 | 1.379 | -0.0284 | No |
| 29 | PXDN |  |  | 4409 | 1.352 | -0.0288 | No |
| 30 | TPM1 |  |  | 4482 | 1.324 | -0.0288 | No |
| 31 | PIK3CA |  |  | 4563 | 1.298 | -0.0296 | No |
| 32 | KIAA0101 |  |  | 4770 | 1.230 | -0.0402 | No |
| 33 | CUTL1 |  |  | 4879 | 1.196 | -0.0435 | No |
| 34 | GDF3 |  |  | 4954 | 1.170 | -0.0444 | No |
| 35 | EFNB2 |  |  | 5094 | 1.118 | -0.0503 | No |
| 36 | NDUFB8 |  |  | 5231 | 1.071 | -0.0563 | No |
| 37 | JARID1B |  |  | 5264 | 1.060 | -0.0544 | No |
| 38 | SLC17A1 |  |  | 5440 | 0.997 | -0.0636 | No |
| 39 | GP9 |  |  | 5512 | 0.970 | -0.0650 | No |
| 40 | TMEPAI |  |  | 6257 | 0.729 | -0.1184 | No |
| 41 | DSCAM |  |  | 6437 | 0.671 | -0.1292 | No |
| 42 | A2BP1 |  |  | 6464 | 0.663 | -0.1285 | No |
| 43 | ADCY1 |  |  | 6582 | 0.625 | -0.1348 | No |
| 44 | NPHS2 |  |  | 7145 | 0.438 | -0.1755 | No |
| 45 | RAVER2 |  |  | 7307 | 0.381 | -0.1862 | No |
| 46 | SETD2 |  |  | 7379 | 0.353 | -0.1901 | No |
| 47 | HSPA1B |  |  | 7563 | 0.283 | -0.2028 | No |
| 48 | SDHA |  |  | 7721 | 0.223 | -0.2138 | No |
| 49 | RSL1D1 |  |  | 8205 | 0.033 | -0.2502 | No |
| 50 | TJP1 |  |  | 8525 | -0.091 | -0.2740 | No |
| 51 | OLFM1 |  |  | 8564 | -0.107 | -0.2764 | No |
| 52 | KCNK2 |  |  | 9014 | -0.317 | -0.3091 | No |
| 53 | THBS1 |  |  | 9418 | -0.522 | -0.3375 | No |
| 54 | SULF1 |  |  | 9912 | -0.791 | -0.3716 | No |
| 55 | PCCB |  |  | 9933 | -0.801 | -0.3698 | No |
| 56 | DYRK1B |  |  | 10141 | -0.926 | -0.3817 | No |
| 57 | RPS6KB1 |  |  | 10180 | -0.944 | -0.3808 | No |
| 58 | XIST |  |  | 10298 | -1.029 | -0.3855 | No |
| 59 | ARID5B |  |  | 10415 | -1.097 | -0.3898 | No |
| 60 | C10ORF76 |  |  | 10496 | -1.160 | -0.3911 | No |
| 61 | CTNND2 |  |  | 10581 | -1.227 | -0.3925 | No |
| 62 | EXOC3 |  |  | 10854 | -1.432 | -0.4073 | Yes |
| 63 | NRCAM |  |  | 10872 | -1.443 | -0.4027 | Yes |
| 64 | JAK1 |  |  | 10903 | -1.475 | -0.3990 | Yes |
| 65 | MCPH1 |  |  | 10942 | -1.522 | -0.3957 | Yes |
| 66 | SLC24A3 |  |  | 11161 | -1.713 | -0.4052 | Yes |
| 67 | PTPN3 |  |  | 11192 | -1.739 | -0.4004 | Yes |
| 68 | HOOK1 |  |  | 11244 | -1.780 | -0.3970 | Yes |
| 69 | GDAP1 |  |  | 11274 | -1.807 | -0.3919 | Yes |
| 70 | HISPPD1 |  |  | 11479 | -2.011 | -0.3992 | Yes |
| 71 | PLK2 |  |  | 11632 | -2.151 | -0.4019 | Yes |
| 72 | RAMP1 |  |  | 11680 | -2.202 | -0.3965 | Yes |
| 73 | COQ4 |  |  | 11693 | -2.213 | -0.3885 | Yes |
| 74 | NR2F2 |  |  | 11694 | -2.214 | -0.3795 | Yes |
| 75 | ISOC1 |  |  | 11714 | -2.233 | -0.3718 | Yes |
| 76 | CACNG4 |  |  | 11858 | -2.378 | -0.3730 | Yes |
| 77 | DNAJC1 |  |  | 11916 | -2.452 | -0.3673 | Yes |
| 78 | CA2 |  |  | 11917 | -2.453 | -0.3574 | Yes |
| 79 | POMT1 |  |  | 11992 | -2.547 | -0.3526 | Yes |
| 80 | MAP3K1 |  |  | 11996 | -2.557 | -0.3425 | Yes |
| 81 | SLK |  |  | 12019 | -2.582 | -0.3337 | Yes |
| 82 | ADIPOR2 |  |  | 12026 | -2.591 | -0.3236 | Yes |
| 83 | NUMA1 |  |  | 12034 | -2.614 | -0.3135 | Yes |
| 84 | SERPINA5 |  |  | 12084 | -2.680 | -0.3063 | Yes |
| 85 | ULK1 |  |  | 12175 | -2.810 | -0.3017 | Yes |
| 86 | SCGB2A1 |  |  | 12325 | -2.994 | -0.3008 | Yes |
| 87 | TIMP3 |  |  | 12387 | -3.085 | -0.2929 | Yes |
| 88 | GPX4 |  |  | 12424 | -3.141 | -0.2829 | Yes |
| 89 | SEMA3C |  |  | 12485 | -3.241 | -0.2743 | Yes |
| 90 | RAB31 |  |  | 12488 | -3.244 | -0.2612 | Yes |
| 91 | PRLR |  |  | 12562 | -3.361 | -0.2531 | Yes |
| 92 | ELOVL2 |  |  | 12695 | -3.604 | -0.2484 | Yes |
| 93 | GPC3 |  |  | 12707 | -3.629 | -0.2345 | Yes |
| 94 | SLC38A1 |  |  | 12718 | -3.652 | -0.2205 | Yes |
| 95 | ZNF217 |  |  | 12755 | -3.756 | -0.2079 | Yes |
| 96 | FAM102A |  |  | 12880 | -4.089 | -0.2007 | Yes |
| 97 | NBEA |  |  | 12894 | -4.140 | -0.1849 | Yes |
| 98 | LAMA5 |  |  | 12907 | -4.167 | -0.1688 | Yes |
| 99 | AGR2 |  |  | 12930 | -4.272 | -0.1532 | Yes |
| 100 | UBL3 |  |  | 12978 | -4.412 | -0.1388 | Yes |
| 101 | NAT1 |  |  | 13126 | -4.922 | -0.1299 | Yes |
| 102 | HRASLS3 |  |  | 13176 | -5.210 | -0.1125 | Yes |
| 103 | IGFBP4 |  |  | 13227 | -5.654 | -0.0933 | Yes |
| 104 | GSTM3 |  |  | 13252 | -5.994 | -0.0708 | Yes |
| 105 | UGDH |  |  | 13266 | -6.157 | -0.0467 | Yes |
| 106 | FLJ20152 |  |  | 13272 | -6.223 | -0.0218 | Yes |
| 107 | C6ORF211 |  |  | 13274 | -6.248 | 0.0035 | Yes |
Table: GSEA details [plain text format]

  

Fig 2: GH\_AUTOCRINE\_DN: Random ES distribution      
 Gene set null distribution of ES for **GH\_AUTOCRINE\_DN**
